# Ecological Health and Freshwater Pathogen Using eDNA Metabarcoding: A Preliminary Assessment for Environmental Surveillance Development in Malaysia

**DOI:** 10.3390/microorganisms13092055

**Published:** 2025-09-04

**Authors:** Jiao Yang, Subha Bhassu, Ghazanfer Ali, Thenmoli Govindasamy, Muhamad Afiq Aziz, Arutchelvan Rajamanikam

**Affiliations:** 1 Animal Genetics and Genome Evolutionary Laboratory (AGAGEL), Department of Genetics and Microbiology, Institute of Biological Sciences, Faculty of Science, Universiti Malaya, Kuala Lumpur 50603, Malaysia; 22088751@siswa.um.edu.my (J.Y.); s2125068@siswa.um.edu.my (G.A.); 22061581@siswa.um.edu.my (T.G.); 2 Virology Laboratory, Department of Genetics and Microbiology, Institute of Biological Sciences, Faculty of Science, Universiti Malaya, Kuala Lumpur 50603, Malaysia; afiqaziz@um.edu.my; 3 Emerging Pathogen Laboratory 1, Department of Parasitology, Faculty of Medicine, Universiti Malaya, Kuala Lumpur 50603, Malaysia

**Keywords:** eDNA, sequencing, metabarcoding, pathogen screening, biodiversity

## Abstract

River water enters human life in various ways, with many disease outbreaks closely linked to contaminated sources. This study collected water samples from the Perak River in Malaysia, extracted environmental DNA (eDNA), and analyzed biological communities using metabarcoding and sequencing techniques to assess the local environmental health of the river. Through 16S rRNA sequencing, 4045 bacterial OTUs were identified, while 18S rRNA sequencing revealed 3422 eukaryotic OTUs, highlighting the diverse microbial and eukaryotic communities in the Perak River. The results showed certain organisms such as *Serratia marcescens* and *Strombidium* with potentially abnormal abundance, based on comparisons with other studies, suggesting possible organic and heavy metal pollution. Additionally, 35 potential pathogens, including bacteria, fungi, and parasites, were detected in the samples, all of which pose potential threats to human and animal health. While most bacterial pathogens are opportunistic, their potential risks should not be overlooked. These findings provide valuable insights into the river’s ecological status and help guide targeted conservation, surveillance and pollution management strategies. Ultimately, this study highlights environmental health issues through biodiversity analysis and identifies pathogens, contributing to the protection of human and animal health and aligning with the principles of the One Health approach.

## 1. Introduction

Freshwater resources in Malaysia are crucial for supporting both the ecosystem and the country’s economy, as they are essential for drinking, agriculture, industry, and sustaining biodiversity [1,2]. Malaysia’s rivers, lakes, and reservoirs are heavily relied upon, providing a primary water source for a significant portion of the population [1,2]. However, despite their importance, freshwater bodies have not been adequately monitored for potential pathogens. This lack of comprehensive monitoring poses a public health risk, as waterborne diseases can spread through contaminated water, impacting communities that depend on these resources for daily use. Increased focus on water quality surveillance, particularly for pathogenic microorganisms, is essential to ensure safe water access and maintain ecosystem health.

The Perak River, which is the second longest river in Malaysia, is referred to as the “River of Life” in the Perak State. It accounts for about 70% of the area of Perak and is also the major source of raw water in this state [3,4]. The Perak River basin has undergone notable land-use changes from 2000 to 2020, with forested areas declining by 20.55% during this period, while urban and agricultural regions have steadily expanded. By 2020, agricultural land alone accounted for approximately 42.46% of the total area [4]. These changes highlight the growing environmental pressures on the Perak River, emphasizing the need for close monitoring of its health as urbanization and agricultural activities continue to intensify.

Waterborne pathogens represent a significant public health challenge and also impacts the economy, agriculture, and food safety. They are primarily transmitted through contaminated freshwater [5] where pathogens that are introduced into water through human and/or animal excreta, survives from several days up to a year [6]. Drinking water, recreational activities, and agricultural irrigation systems serve as pathways through which humans may be exposed to pathogens [5,6]. Approximately 1 million people, including around 395,000 children die each year from diarrhea-related diseases caused by unsafe drinking water and poor sanitation [7] (WHO, 2019). There are also economic problems associated with waterborne diseases where come countries are burdened by medical costs due waterborne diseases such as in the United States with 3.33 billion dollars spent annually [8]. The use of contaminated irrigation water can lead to foodborne illnesses such as salmonellosis, with an estimation of 10 percent of the global population consuming crops irrigated with such water [9,10,11].

A healthy river system contributes to ecological health, economic activity, and social well-being [12]. Biodiversity plays an important role in providing ecological services, such as water purification, water quality maintenance, and nutrient cycling in aquatic environments. Microorganisms are key for assessing the health of river systems and overall ecological integrity [12,13,14,15]. However, to date, there are no established techniques or efforts in monitoring microbial diversity in Malaysian freshwater. However, to date, there are no established techniques or efforts in monitoring microbial diversity in Malaysian freshwater. Traditional monitoring approaches, which can be labor-intensive and time-consuming, and often involve measuring multiple physicochemical parameters, such as nutrient concentrations (e.g., ammoniacal nitrogen (NH_4_^+^-N), nitrate nitrogen (NO_3_^−^-N), ortho-phosphate phosphorus (PO_4_^3−^-P)), heavy metals (e.g., iron (Fe), manganese (Mn), magnesium (Mg)), and general water quality indicators (e.g., pH, hardness, dissolved oxygen (DO)) [16,17]. Additionally, microbial culture methods, a common approach for pathogen detection, may fail to identify rare or unculturable microorganisms, leading to incomplete assessments [18,19]. These limitations highlight the need for more comprehensive and efficient tools like eDNA metabarcoding, which can offer a holistic view of microbial communities. It is crucial to preserve river ecosystems and safeguard human and wildlife health.

Recent technological advancements have enabled and improvised high-throughput detection of organism from environmental samples. Environmental DNA (eDNA) combined with next-generation sequencing and meta-barcoding technologies significantly improves the accuracy and efficiency of biodiversity monitoring [20,21]. This method is reportedly more time- and cost-efficient, as well as sensitive and non-invasive compared to traditional sample collection techniques, making it a suitable candidate for freshwater ecosystem studies [22,23]. Although this approach aids in tracking waterborne pathogens, particularly those that are difficult to culture [24], there are only sporadic studies to assess its use in detecting freshwater pathogen and ecologic health.

In this study, we explored eDNA metabarcoding, targeting the 16S rRNA and 18S rRNA genes, to assess site-specific bacterial and eukaryotic diversity in Malaysia. We conducted a preliminary assessment of river pollution, ecological health and its association with potential pathogens. This study serves as a valuable case for advancing the application of eDNA metabarcoding in public health protection. Additionally, the findings also highlight its potential to inform water resource management policies and guide the implementation of targeted pollution control strategies.

## 2. Materials and Methods

### 2.1. Study Area and Sample Collection

**Figure 1:**
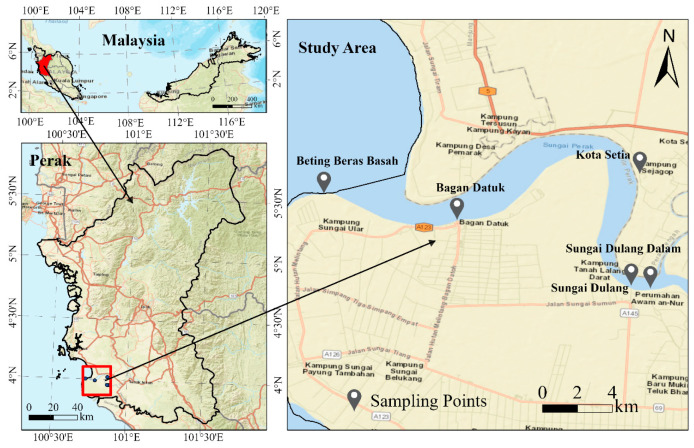
Study map showing the sampling locations (Created by ArcGis Pro).

Our pilot study was conducted within approximately 30 km of the downstream section of the Perak River (Perak, Malaysia). The downstream section of a river often accumulates biological information from the entire river, providing a more comprehensive understanding of its biodiversity with limited sampling points [25]. This approach also saves both sampling time and research costs. Sterile 1 L bottles were used for sampling to minimize contamination. Water samples were collected from 5 locations at the downstream of the Perak River, each area 3 sampling points (Figure 1 and Table 1). Five liters of water were collected in bottles and about 1 L for eDNA extraction. The total number of the sample size was 15. In addition, 1 L of distilled water was used as a negative control.

### 2.2. Extraction of eDNA

eDNA may degrade rapidly within 1–2 days [26], so it is recommended to filter and preserve the samples within 24 h [27]. In our research the collected water samples were promptly transported to the laboratory and processed within 12 h to minimize DNA degradation. A 0.45 µm cellulose nitrate membrane (Bioflow, Selangor, Malaysia) was used to filter the samples with an oil-free vacuum pump (Rocker 300, Taiwan, China). The filter was then ground into powder with liquid nitrogen and stored at −20 °C until DNA extraction. Filter cups, grinding utensils, forceps and centrifuge tubes involved in the process were all autoclaved.

eDNA was extracted using the phenol-chloroform-isoamyl (PCI) method and the extraction process was performed in sterile laminar flow cabinets. First, 100 µL of lysis buffer (50 mM Tris, 150 mM NaCl, 1% Triton, 5% glycerol, pH 8), 100 µL of 10% SDS solution, and 20 µL of proteinase K were added to a 1.5 mL centrifuge tube containing filter paper fragments. The mixture was briefly vortexed and incubated at 65 °C for 30 min. After incubation, it was vortexed again and centrifuged at 5000 rpm for 5 min at 4 °C. An equal volume of phenol/chloroform/isoamyl (PCI) solution was then added, and the mixture was centrifuged at 12,000 rpm for 10 min at 4 °C. The supernatant was transferred, and an equal volume of chloroform/isoamyl (CI) solution was added, mixed well, and the mixture was centrifuged at 12,000 rpm for 10 min at 4 °C. The supernatant was transferred again, and 0.1 volume of 5 M NaCl and 2 volumes of absolute ethanol were added, followed by vigorous shaking to mix. The mixture was then placed at −20 °C overnight. The precipitate was centrifuged at 12,000 rpm for 15 min at 4 °C. The supernatant was discarded, and the precipitate was washed with 70% ethanol, followed by centrifugation at 12,000 rpm for 15 min at 4 °C. The supernatant was discarded again, and the procedure was repeated once more. The centrifuge tube was then air-dried to allow the ethanol to fully evaporate. Finally, 50 µL of TE buffer preheated to 65 °C was added, mixed thoroughly, and the eDNA extraction was completed. The sample was stored at −20 °C until further analysis.

### 2.3. PCR Amplification and Sequencing

The extracted DNA was subjected to PCR amplification for community analysis. The 16S rRNA and 18S rRNA genes have been widely used in prokaryotic (bacteria and archaea) and eukaryotic biodiversity studies and are common genetic markers for phylogenetic and taxonomic analysis [28]. These two genes possess highly conserved sequences along with variable regions, making them suitable for taxonomic studies across a broad range of organisms using universal primers [29,30,31]. The V3-V4 region of the 16S rRNA gene offers higher resolution for identifying lower bacterial taxonomic levels (genus and species) compared to other regions [32]. Similarly, the V4 region of the 18S rRNA gene is frequently applied in eukaryotic studies, capable of detecting a high number of OTUs [33,34,35]. So, in this study, the primers 341F (5′-CCTAYGGGRBGCASCAG-3′) and 806R (5′-GGACTACNNGGGTATCTAAT-3′) [36] were used to amplify the 16S rRNA V3-V4 of bacteria, and the primers TAReuk454FWD1 (5′-CCAGCASCYGCGGTAATTCC-3′) and TAReukREV3 (5′-ACTTTCGTTCTTGATYRA-3′) [37] were used to amplify the 18S rRNA V4 region of eukaryotes. The reactions were performed in a 20 µL system containing 4.0 µL of 5× buffer, 2 µL of 2.5 mM dNTPs, 0.8 µL of each primer (5 µM), 0.4 µL of FastPfu Polymerase, and 10 ng of template DNA, with the remaining volume made up with double-distilled water. Firstly, eDNA was diluted 30-fold with nuclease-free water to achieve a final concentration of approximately 10 ng/μL. This dilution step was performed to reduce the inhibitory effects of environmental substances, such as humic acids, on PCR amplification, improving both the success rate of the PCR reaction and data quality [38]. The PCR conditions for both genes were as follows: 95 °C for 2 min, followed by 25 cycles at 95 °C for 30 s, 55 °C for 30 s, and 72 °C for 30 s, with a final extension at 72 °C for 5 min.

**Figure 2:**
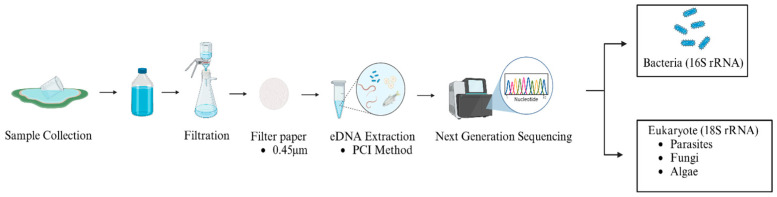
Workflow of the experiment.

PCR products were identified using 2% agarose gel electrophoresis, then purified with the AxyPrep DNA Gel Extraction Kit (Axygen Biosciences, Union City, CA, USA), and quantified using QuantiFluor™-ST (Promega, Madison, WI, USA). Finally, sample libraries were pooled in equimolar amounts and sequenced using paired-end (2 × 250/300 bp) on an Illumina MiSeq platform, following standard protocols. The workflow of the experiment is shown in Figure 2.

### 2.4. Data Analysis

Raw data were stored using fastaq format and then demultiplexed for quality filtering using Quantitative Insights into microbial Ecology (version 1.9.1). Truncated 300 bp reads of any locus with an average quality score of less than 20, and discarded reads shorter than 50 bp. Barcodes must be matched exactly, and primer matching allows for up to 2 mismatched nucleotides. Removed reads containing ambiguous characters, reorganize only sequences that overlap by more than 10 bp, reads which failed to reorganize were discarded.

The taxonomy of each 16S rRNA gene sequence was analyzed by RDP Classifier (Version 2.2, http://sourceforge.net/projects/rdp-classifier/ (accessed on 10 October 2024)) against the Silva (SSU123) 16S rRNA database using a confidence threshold of 0.7. The taxonomy of each 18S rRNA gene sequence was analyzed by RDP Classifier (Version 2.2, http://sourceforge.net/projects/rdp-classifier/ (accessed on 10 October 2024)) against the Silva (SSU123) 18S rRNA database using a confidence threshold of 0.7. The microbial community could be used to compare similarity or dissimilarity between different sample groups. Operational Units (OTUs) were clustered with 97% similarity cutoff using Usearch (version 10, http://drive5.com/uparse/ (accessed on 12 October 2024)). Sparse analysis at the OTU level was performed using mothur (version v.1.30.1, http://www.mothur.org/), and indices such as Observed species, Chao1, ACE, Shannon, Simpson, Pielou’s evenness, and Faith’s Phylogenetic Diversity were calculated to assess alpha diversity. Principal Coordinate Analysis (PCoA) was conducted to evaluate beta diversity, based on the Unweighted UniFrac distance and PERMANOVA statistical method. Finally, metagenomic analysis results were visualized using R Studio (version 4.4.1).

## 3. Results

### 3.1. Illumina MiSeq Data Supports Sufficient Microbial Diversity Analysis

The raw data generated from the Illumina MiSeq platform were quality-filtered using QIIME (version 1.9.1), resulting in 918,194 sequences for 16S and 729,490 sequences for 18S rRNA (Supplementary Table S1), which were then used for subsequent OTU clustering analysis. Among the sequences, 98.13% of the 16S rRNA sequences were primarily between 301–400 bp in length, while 99.18% of the 18S sequences were concentrated between 401–500 bp (Supplementary Table S2). Negative control showed no significant reads. The eDNA from Location 5 was excluded from 18S rRNA sequencing analysis as it was anomalous. Nevertheless, analysis was conducted from the 12 sampling points especially for 18S rRNA-related data.

**Figure 3:**
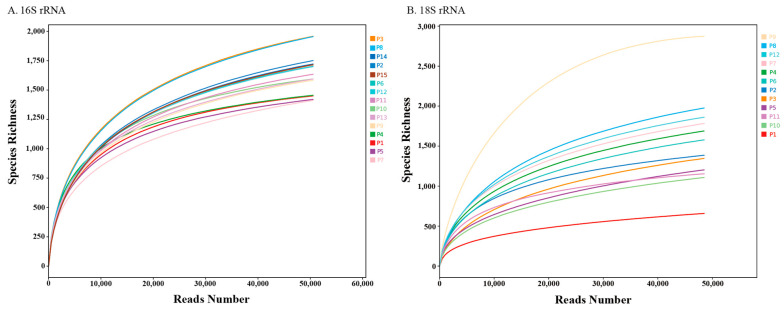
Rarefaction curve highlights the relationship between the number of read samples and observed OTUs for (**A**) 16S rRNA and (**B**) 18S rRNA sequencing data.

After OTU clustering analysis, 4045 OTUs for 16S rRNA and 3422 OTUs for 18S rRNA (Supplementary Table S3) were generated and used for subsequent analysis. Figure 3 illustrates rarefaction curves, with the *x*-axis showing sequencing depth as the number of sequencing reads, and the *y*-axis representing species richness as the observed number of OTUs. As the sequencing depth increases, the growth in species richness gradually slows, exhibiting a clear trend toward stabilization. This pattern suggests that the current sequencing effort adequately captured the microbial diversity at each sampling site, and further sequencing is unlikely to yield a significant increase in detected OTUs. This indicates that the sample size and sequencing depth in this study are likely sufficient to represent the microbial diversity in the environment. Therefore, the sequencing data offers a reliable basis for subsequent analyses.

### 3.2. Taxonomic Composition of eDNA Metabarcoding Data

**Figure 4:**
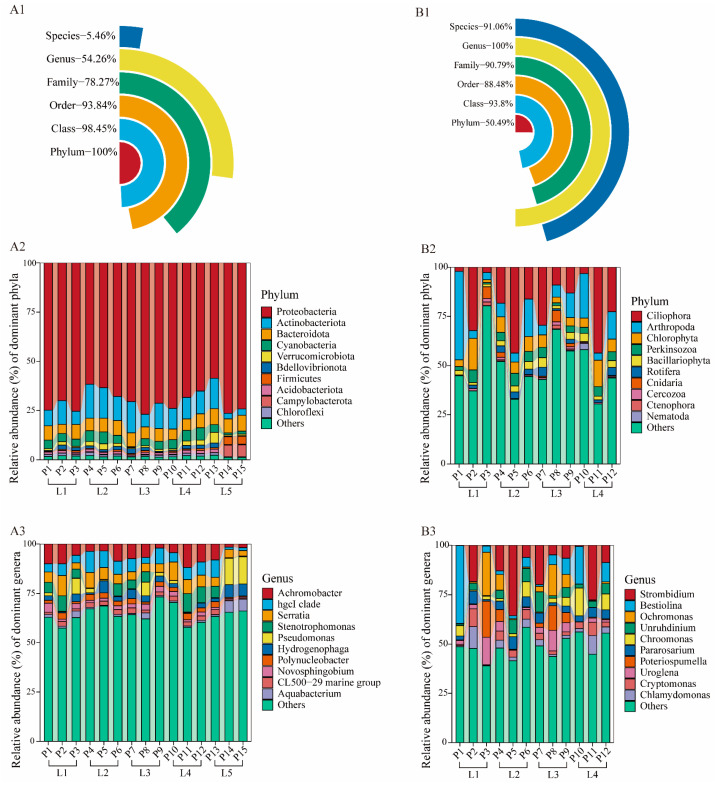
Overview of OTU Taxonomic Annotation. A: 16S rRNA results; B: 18S rRNA results. The coverage of sequence annotations at different taxonomic levels (**A1**,**B1**). Taxonomic composition and relative abundances of major microbial communities at the phylum (**A2**,**B2**) and genera (**A3**,**B3**) level in each water sample (Note: Microorganisms whose percentage less than 1% are included in “others”.).

All OTUs of 18S rRNA and 16S rRNA were able to be annotated (Figure 4(A1,B1)), and reached 100% annotation coverage at the phylum and genus levels, respectively. In addition, 16S rRNA sequences (Figure 4(A1)) were well annotated at higher taxonomic levels, such as order and phylum, achieving an annotation rate of over 90%. However, at lower taxonomic levels, particularly at the species level, the annotation coverage was significantly lower, reaching only 5.46%. 18S rRNA (Figure 4(B1)) performed well in annotation coverage at every taxonomic level except phylum, almost 90% of OTUs can be annotated at these taxonomic levels. In the end, for the 16S rRNA sequencing result, bacteria were assigned to 42 phyla, 85 orders, 241 orders, 348 families, 650 genera, and 194 species. For the 18S rRNA sequencing result, eukaryotes were assigned to 38 phyla, 107 orders, 248 orders, 418 families, 708 genera, and 1105 species.

The taxonomic microbial composition and relative abundance at the phylum and genera levels for each sample are shown in Figure 4. Across all samples, Proteobacteria undoubtedly held a dominant position among bacteria at the phylum level (Figure 4(A2)). Among eukaryotes, Ciliophora was the dominant group, followed by Arthropoda and then Chlorophyta (Figure 4(B2)). At the genus level, *Achromobacter*, *hgcI clade*, and *Serratia* are dominant among bacteria (Figure 4(A3)), while *Strombidium*, *Bestiolina*, and *Ochromonas* are the most prevalent among eukaryotes (Figure 4(B3)).

**Figure 5:**
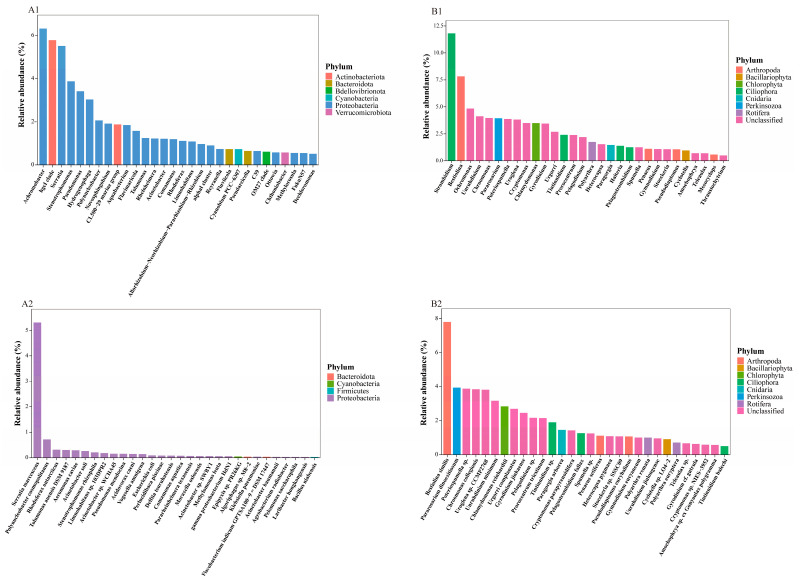
Dominant Genus and Species in 16S and 18S rRNA Data. A:16S rRNA results; B 18S rRNA results. The top 30 microorganisms at genus (**A1**,**B1**) and species level (**A2**,**B2**) are selected, the color of the bar indicates the taxonomic information at phylum level.

Among bacteria, most of the dominant genera (Figure 5(A1)) and species (Figure 5(A2)) belong to Proteobacteria. *Achromobacter* had the highest relative abundance at genus level, exceeding 6%. Following this were the *hgcI clade* and *Serratia*, both with relative abundances close to 6%. At the species level, *Serratia marcescens* had the highest relative abundance (over 5%), significantly ahead of the second most abundant species, *Polynucleobacter cosmopolitanus*.

Among eukaryotes, at the genus level (Figure 5(B1)), *Strombidium* had a relative abundance of approximately 12%, while the second most abundant genus, *Bestiolina*, accounted for about 7.5%. Most of the other dominant genera were unclassified. At the species level (Figure 5(B2)), the top three identified species were *Bestiolina similis*, *Pararosarium dinoexitiosum*, and *Chlamydomonas reinhardtii*, with *Bestiolina similis* having a relative abundance about twice that of the other two species. Most of the remaining dominant species were also unclassified.

### 3.3. Comparison of Biodiversity Between River Samples

#### 3.3.1. Alpha Diversity

**Figure 6:**
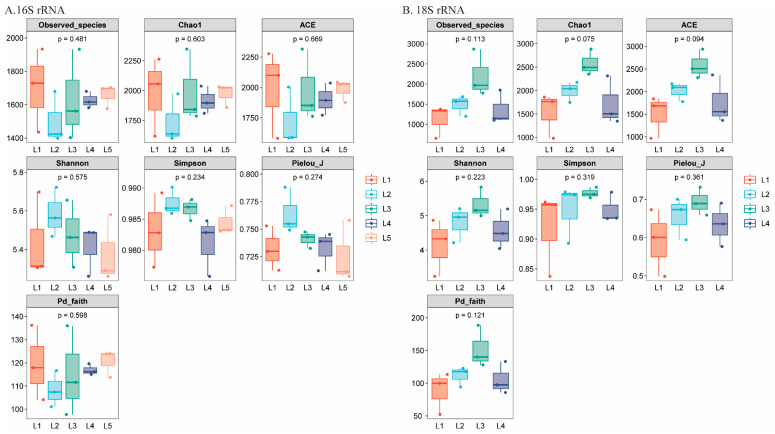
Comparison of alpha diversity values among samples collected in different locations with three sampling points each. (**A**) Alpha diversity for 16S rRNA sequencing data, and (**B**) alpha diversity for 18S rRNA sequencing data. Significance analysis using Kruskal–Wallis test showed no significant differences between all samples (*p* > 0.05).

Firstly, alpha diversity indices were used to analyze species diversity (Figure 6). These included the Observed species, Chao1, and ACE metrics for species richness, Shannon, Simpson, and Pielou’s evenness (Pielou_J) metrics for both richness and evenness, as well as the Faith Phylogenetic Diversity (Pd_faith) metric to measure phylogenetic distances. Species richness indices assess the number of species within a sample, with Observed_species reflecting the directly observed species count. Chao1 and ACE build on this by estimating the total number of species at the sampling site, including those that may not have been directly observed [39,40]. The Shannon and Simpson indices are used to evaluate community diversity, with a higher Shannon index indicating greater species diversity within the sample [39]. The Simpson index is particularly sensitive to the dominance of certain species, making it a useful measure for identifying the presence of dominant taxa within a community. Higher values approaching 1 indicate a community dominated by a few species, resulting in uneven species distribution and relatively lower overall diversity [41]. The Pielou_J measures the uniformity of individual distribution across species within a community, with values closer to 1 indicating a more balanced allocation of individuals among species in terms of their numbers [42]. The Pd_faith index reflects phylogenetic diversity. A higher Pd_faith value indicates that the species within the community are more widely dispersed on the evolutionary tree, suggesting greater phylogenetic diversity [43].

In the 16S rRNA results (Figure 6A), L1 exhibited the highest Observed_species, Chao1, and ACE indices, demonstrating the greatest species richness at this location. The Shannon and Simpson indices for L2 are the highest, indicating that the community at this location has greater diversity but an uneven distribution of species abundance. The species evenness at all locations is relatively moderate, according to the Pielou_J index. In terms of phylogenetic distance, the species in L2 are likely to have closer evolutionary relationships.

In the 18S rRNA results (Figure 6B), the Observed species, Chao1, and ACE indices suggest that L3 has the highest species richness. L3 shows results similar to those of L2 in the 16S rRNA analysis, with higher community diversity and uneven species abundance distribution, as indicated by the Shannon and Simpson indices. Overall, the species evenness at all locations is relatively low, according to the Pielou_J index. In terms of phylogenetic relationships, the genetic distances among species in L3 are closer compared to those at other locations, based on the Pd_faith index.

However, in both the 16S rRNA and 18S rRNA results, the alpha diversity indices show no significant differences among the research area.

#### 3.3.2. Beta Diversity

**Figure 7:**
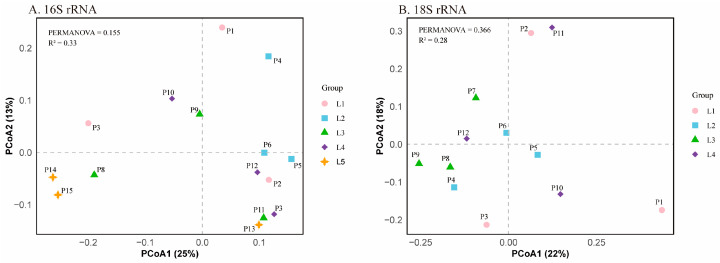
Principal coordinates analysis (PCoA) of communities in each group (L1, L2, L3, L4 and L5) based on Unweighted-Unifrac distance (*p* > 0.05). (**A**) PCoA for 16S rRNA sequencing data with PERMANOVA results (R2 = 0.33). (**B**) PCoA for 18S rRNA sequencing data with PERMANOVA results (R2 = 0.28).

Beta diversity was used to measure variability in species composition among sampling areas in the Perak River (Figure 7). Beta diversity shows the variation in biological community composition between different habitats or sampling locations, providing insights into the spatial heterogeneity of these communities [44]. Among the results of bacterial composition in each region (Figure 7A), P14 and P15 of L5 were similar, while P13 was more different from each other. P5 and P6 from L2 are more similar, while P4 is farther apart, and all other sampling sites are more spread out in Figure 7A, suggesting greater differences in bacterial composition across these locations. In the results of the eukaryotic analysis (Figure 7B), P8 and P9 were closer together in L3, P5 and P6 were closer together in L2, and again the samples from the remaining sites were more dispersed in Figure 7B. This demonstrates the presence of localized similarity at certain sampling sites, though variations in eukaryotic community composition are observed across most regions. Overall, the species composition of both bacteria and eukaryotes, however, did not show significant differences between the sampling sites.

### 3.4. Diversity of Potential Pathogens in Perak River

**Figure 8:**
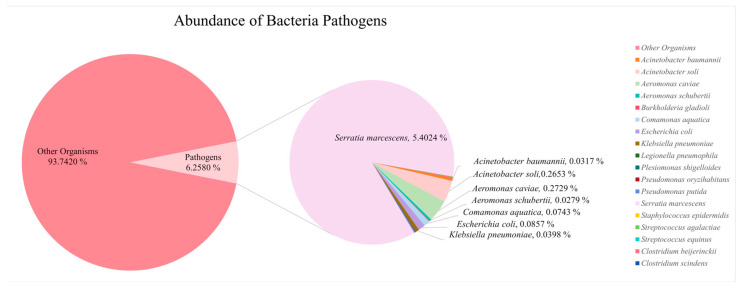
Abundance of Potential Pathogenic Bacteria Detected in Perak River Based on Reads Count.

Reference was made to the database of global catalog of pathogens (https://nmdc.cn/gcpathogen/ (accessed on 16 October 2024)) to screen potential pathogens. A total of 19 bacterial pathogens (Table 2) from the phyla Pseudomonadota and Bacillota were selected based on the results of the 16S rRNA sequence OTUs species annotation, almost all of them posing a threat to human health, especially in populations with compromised immune systems. Among these pathogens, *Serratia marcescens* had 45,649 reads, *Aeromonas caviae* had 2306 reads, and *Acinetobacter soli* had 2242 reads, showing relatively high abundance. Bacterial pathogen richness accounted for about 6.26% of the 16S clustered OTUs, where the specific proportion of individual pathogens is shown in Figure 8 (the too rare species do not show specific data).

Based on the 18S rRNA sequence OTUs species annotation results, seven fungal pathogens (Table 3) and nine parasitic pathogens (Table 4) were screened. *Pythium insidiosum* is not traditionally considered a parasite [45], but it has been classified as a parasitic pathogen in the Global Catalog of Pathogens. Both fungal and parasitic pathogens are potential threats to human health, and the parasitic pathogens identified in Perak River are more likely to be harmful to animals (Table 3 and Table 4). The abundance of these pathogens is generally low. The most abundant fungal pathogen is *Meyerozyma guilliermondii* with 402 reads, while *Eimeria bukidnonensis* is the most abundant parasite with 768 reads. All other pathogens had only a few dozen or even single-digit reads, making them extremely rare. The proportions of fungal and parasitic pathogens are shown in Table 5. Although they only account for 0.2%, their potential pathogenicity should not be overlooked.

**Table 2:** Diversity of potential bacterial pathogens detected in Perak River.

No.	Phylum	Organism	Abundance of Reads	Host	Diseases	References
1	Pseudomonadota	*Acinetobacter baumannii*	268	Humans	Ventilator-associated pneumonia, blood stream infections, urinary tract infections and meningitis	[46,47,48]
2	Pseudomonadota	*Acinetobacter soli*	2242	Humans	Bacteremia	[49,50,51]
3	Pseudomonadota	*Aeromonas caviae*	2306	Aquatic animals such as *Micropterus salmoides*, *Clarias gariepinus*	Decaying and reddening of the body surface, mass deaths, visceral congestion and hepatocellular necrosis	[52,53]
				Humans	Pneumonia, primary bacteremia and biliary tract infections	[54]
4	Pseudomonadota	*Aeromonas schubertii*	236	Aquatic animals such as *Channa striata*, *Oreochromis niloticus*	Near-death state, hemorrhage, white nodules on the liver and inflammation in the organs	[55,56]
5	Pseudomonadota	*Burkholderia gladioli*	38	Plants	Plant tissue decay	[57]
				Humans	Pulmonary infection and systemic abscesses (patient with Cystic Fibrosis)	[57,58]
				Animals	N/A	[57,59]
6	Pseudomonadota	*Comamonas aquatica*	628	Humans	Bacteremia and septic shock	[60]
				*Labeo rohita*	Tail and fin rot	[61]
7	Pseudomonadota	*Escherichia coli*	724	Humans	Bacteremia, hemolytic uremic syndrome (HUS), watery and/or bloody diarrhea, and colitis	[62,63]
8	Pseudomonadota	*Klebsiella pneumoniae*	336	Humans	Urinary tract infections, liver abscesses, pneumonia, meningitis, and bacteremia	[64,65,66]
9	Pseudomonadota	*Legionella pneumophila*	23	Humans	Legionnaires’ disease (LD): pneumonia, fever, respiratory, gastrointestinal and neurological symptoms	[67,68]
10	Pseudomonadota	*Plesiomonas shigelloides*	18	Humans	Diarrhea, gastroenteritis, sepsis, central nervous system diseases, and eye infections, etc.	[69,70]
				Freshwater fish such as Tilapia, *Acipenser dabryanus*	Reduced activity, pale body color, hemorrhages on the body surface, internal organ damage, and high mortality rate	[70,71]
11	Pseudomonadota	*Pseudomonas oryzihabitans*	53	*Cucumis melo* L., rice	Browning and wilting of infected tissues	[72,73]
				Humans	Skin abscess, bacteremia and yellow-green discoloration of the nails	[74,75,76]
12	Pseudomonadota	*Pseudomonas putida*	147	Humans	Skin and soft tissue infections, bacteremia	[77]
				*Oreochromis niloticus*	Skin ulcers, subcutaneous hemorrhages, mild internal organ swelling, and intestinal engorgement	[78]
13	Pseudomonadota	*Serratia marcescens*	45,649	Humans	Urinary tract infections (UTIs), pneumonia, keratitis, conjunctivitis, surgical wound infections, bacteremia, sepsis, and meningitis	[79,80]
				Animals and insects such as honey bees	N/A	[79,81,82]
14	Bacillota	*Staphylococcus epidermidis*	34	Humans	Late-onset neonatal sepsis	[83]
15	Bacillota	*Streptococcus agalactiae*	4	Tilapia	Meningitis	[84]
				Humans	Bacteremia, pneumonia and chorioamnionitis	[85,86]
16	Bacillota	*Streptococcus equinus*	36	Humans	Infective endocarditis (IE), brain abscess	[87,88]
19	Bacillota	*Enterococcus* sp.	35	Humans	Endocarditis, sepsis, meningitis, bacteremia, urinary tract infections,	[89,90,91]

**Table 3:** Diversity of potential fungal pathogens detected in Perak River.

No.	Phylum	Organism	Abundance of Reads	Host	Diseases	References
1	Ascomycota	*Acremonium* sp. 1 FW2SW3	2	Humans	Infection peritonitis, fungal osteomyelitis	[92,93]
2	Ascomycota	*Meyerozyma guilliermondii*	402	Humans	Bloodstream infections and fever	[94,95,96]
3	Ascomycota	*Pichia kudriavzevii*	11	Humans	Late-onset sepsis, histopathologic changes in the intestine, mycosis peritonitis	[97,98,99]
4	Ascomycota	*Wickerhamomyces anomalus*	2	Humans	Keratitis, candidemia, meningitis, fungemia, ventriculitis, keratitis and endophthalmitis	[100,101,102]
5	Basidiomycota	*Cryptococcus* sp. SJ8L05	8	Humans	Pneumonia, meningitis	[103,104,105]
6	Basidiomycota	*Naganishia albida*	15	Humans	Skin lesions, otomycosis	[106,107]
7	Basidiomycota	*Rhodotorula mucilaginosa*	49	Humans	Meningitis	[108,109,110]

**Table 4:** Diversity of potential parasite pathogens detected in Perak River.

No.	Phylum	Organism	Abundance of Reads	Host	Diseases	References
1	Apicomplexa	*Babesia* sp. Kh-Hj540	3	Brown bear	N/A	[111,112,113]
2	Apicomplexa	*Blastocystis* sp.	3	Humans	Diarrhea, dermatological symptoms	[114,115,116]
				Animals	N/A	[114,117]
3	Apicomplexa	*Cryptosporidium serpentis*	21	Reptiles	High mortality	[118,119]
4	Apicomplexa	*Eimeria bukidnonensis*	768	Cattle	Bovine eimeriosis (intestinal disease)	[120,121]
5	Apicomplexa	*Eimeria* sp.	5	Livestock	Diarrhea, anorexia and death	[122,123]
6	Apicomplexa	*Theileria* sp.	7	Livestock and wild animals	Fever, hemoglobinuria, anemia, and death	[124,125,126]
7	Myzozoa	*Perkinsus olseni*	7	Molluscs	Tissue inflammation, impaired reproduction, reduced growth and mass mortalities	[127,128,129]
8	Myzozoa	*Perkinsus qugwadi*	50	Molluscs	Mass mortalities	[130]
9	Oomycota	*Pythium insidiosum*	25	Animals	Pythiosis	[131,132]
				Humans	Keratitis	[133]

N/A: No relevant information is currently available in the existing literature or reports.

**Table 5:** Abundance of Potential Pathogenic Fungi and Parasites Detected in Perak River Based on Reads Count.

Pathogens	Percentage (in All OTUs)	Organisms	Reads	Percentage (in All Pathogens)
Fungi	0.0706%	*Meyerozyma guilliermondii*	402	29.17%
		Other fungal pathogens	87	6.31%
Parasites	0.1283%	*Eimeria bukidnonensis*	768	55.73%
		Other parasite pathogens	121	8.78%

## 4. Discussion

In this pilot study, the eDNA meta barcoding using NGS technique was used to monitor biodiversity, community composition, and potential pathogens in the Perak River, Malaysia, in order to assess the ecological health of the river system and its potential impact on public health.

### 4.1. Biodiversity Helps Initial Assessment of Ecological Health

The health of an aquatic ecosystem can be initially assessed by its biodiversity, as a diverse community composition often indicates a relatively stable ecosystem [134]. In particular, microbial diversity is highly sensitive to pollution, with bacteria often playing key roles in nutrient cycling and water purification [135,136]. Monitoring bacterial diversity can therefore quickly reveal the health of ecosystems [135,136]. The alpha diversity index can be used to evaluate environmental health. Shannon’s index values below 1 indicate that the water body is highly polluted, values between 1 and 2 suggest moderate pollution, while values above 3 indicate a stable environment, reflecting higher species richness [137,138]. Additionally, the closer Simpson’s and Pielou’s evenness indices are to 1, the fewer dominant species there are, and the more evenly distributed the species become [139]. Therefore, based on the biodiversity indices in this study (Shannon’s index 3.23–5.26, Simpson’s index 0.83–0.98, Pielou’s evenness index 0.49–0.78), it can be inferred that the Perak River is relatively ecologically stable, but the overall species distribution is only moderately even. In the past, these indices have been widely used to assess the health of aquatic ecosystems, such as the Chambal River in India [134], the Banas River Corridor in India [139], Taylor Creek in Nigeria [135], and Wular Lake in Kashmir [138], and others. The results of this study showed no significant differences in alpha diversity across all sampling sites, suggesting that eukaryotic and bacterial diversity is relatively homogeneous in the study area. This may be due to the small size of the study area or, similar to the findings of Luiz et al.’s study on the Sinos River (Brazil) in winter [140], the Perak River may have a long stretch of watershed with more homogeneous biodiversity.

Another important metric, the beta diversity index, is used to reveal differences in community structure between sampling sites [141]. However, in this study, all sampling sites exhibited similar β-diversity with no significant differences, indicating a high degree of similarity in species composition across the sites. This is likely due to the small size of the study area, low variability in environmental factors (such as water temperature, water quality, habitat, and climate), stable water connectivity, minimal environmental disturbance, and the greater adaptability of the biological community to these conditions [142,143,144]. For example, Zhang et al. found no significant differences in the beta diversity of phytoplankton across the spatial and temporal distributions of subtropical rivers in China [143]. Similarly, Liu et al. demonstrated that species turnover was not significant in the phytoplanktonic microbial communities across different regions of the Laptev Sea and Lena River Estuary, showing similar beta diversity [145]. Heino et al. reported comparable findings for invertebrate communities in different streams within the Oulanka River Basin in northeastern Finland [146].

The observed similarity in beta diversity in this study can be further attributed to the high water connectivity within the study area. In the L1-L5 river section, there are no significant tributary inflows or barriers that could disrupt the connectivity of the river water. Interconnected water systems allow species to migrate between different locations, leading to homogenization of microbial community composition. In such systems, minimal habitat isolation reduces the opportunity for microbial communities to proliferate at specific sites. Previous studies have also indicated that high connectivity often leads to minimal significant differences in beta diversity. Additionally, the sampling area in this study was limited to approximately 30 km of river stretch. Within this relatively narrow environmental range, water temperature and nutrient levels exhibited limited variation across different sites, resulting in a stable and uniform environment. Stable conditions tend to support microbial communities with similar compositions, as they reduce selective pressures for site-specific adaptations. As highlighted by Da Silva et al., species differences are smaller in stable environments, leading to more similar beta diversity [147]. Human activities may also be a potential influencing factor. Agricultural runoff and minor urban wastewater discharge could lead to nutrient enrichment, reducing the natural dynamics and heterogeneity of the river [148,149]. These human activities can obscure natural environmental gradients, resulting in more uniform biological communities. For example, dam construction suppresses the seasonal flow variations of rivers [150], while pollutants from agricultural runoff become the main contributors to river nutrients [151], leading to habitat homogenization, which may ultimately result in a lack of significant beta diversity differences. Combined with the alpha diversity analysis, which indicates that the study area is not severely polluted, the similar beta diversity observed in this study is more likely attributed to the relatively small size of the study area and the high connectivity of the river system.

Overall, based on the results of the bacterial and eukaryotic biodiversity analysis, we can preliminarily infer that the Perak River in this study area is ecologically stable, with low levels of pollution and minimal external interference. In the future, expanding the sampling area and incorporating environmental parameters will allow for a more systematic assessment of biodiversity dynamics and the overall health of the river system.

### 4.2. Influence of Sampling Limitations on Diversity Analysis

Due to the missing sample data from Location 5 in the 18S rRNA sequencing results, concerns may arise regarding the accuracy and reliability of our findings. However, both the 16S rRNA sequencing results across all five sampling sites and the 18S rRNA results from four sites showed no significant differences in alpha and beta diversity. This suggests that the inclusion of Location 5 would likely not alter the overall findings, with minimal impact on the interpretation of community structure.

Moreover, previous research has shown that downstream sampling effectively reflects the biodiversity of an entire river system, as downstream sites integrate biological and ecological signals from upstream regions [25]. In this study, all sampling points are located at the downstream end, which already provides a representative overview of the river’s microbial and eukaryotic communities. The high water connectivity and stable environmental conditions within the study area further reduce spatial variation, enhancing the representativeness of the sampled sites.

Thus, while one sampling location is missing, the consistent diversity results and strategic downstream sampling ensure that the findings remain reliable and accurately reflect the ecological dynamics of the river system.

### 4.3. Bacterial Community Composition Indicative of Ecological Pollution

In the bacterial community distribution at the lower end of the Perak River, Proteobacteria occupies the lion’s share and is considered the dominant taxon in freshwater ecosystems [152]. Following Proteobacteria are Actinobacteria and Bacteroidota, which also have relatively high proportions. The results are consistent with many studies on other rivers and lakes, where these taxa are typically dominant in most aquatic environments, such as the Nanfei River [153], Songhua River [154], and Seine River [155].

Proteobacteria are well known for their role in the degradation of organic matter and their correlation with nutrient levels in water bodies [156]. They are also often associated with various forms of pollution, including metal contamination, fecal pollution, and organic matter pollution [153,157]. Among them, Gammaproteobacteria is typically not a dominant group in most aquatic environments [158], but in this study, its relative abundance ranged from 40.8% to 70.5%. It is usually abundant in heavily polluted water bodies and is significantly correlated with concentrations of heavy metals (e.g., nickel, cobalt, cadmium), as well as nitrogen and phosphorus pollution [152,158,159]. Its higher abundances typically indicate more severe pollution [152,158,159]. The second most abundant group was Alphaproteobacteria (relative abundance 5.7–17.9%), which also increased with pollution levels. However, in heavily polluted areas, its abundance declined, showing an opposite trend to Gammaproteobacteria [158]. Both *Achromobacter* and *Serratia* were dominant in all samples and belonged to Proteobacteria. However, at the species level, *Serratia marcescens* (mean relative abundance > 5%) showed absolute dominance, while Achromobacter’s dominance was mainly attributed to the number of its species, rather than the abundance of a single species. *S. marcescens* is widely distributed in the environment, characterized as a resistant Gram-negative bacterium with pathogenic potential [160]. It has been reported to exhibit significant tolerance to heavy metals and can effectively adsorb or immobilize environmental heavy metals such as lead (Pb), cadmium (Cd), and chromium (Cr) through various metabolic pathways [160]. Moreover, *S. marcescens* has organophosphorus hydrolase, which can convert complex organophosphorus compounds into absorbable inorganic phosphorus. Since this enzyme is uncommon in other bacteria, *S. marcescens* has a strong competitive advantage in organically polluted environments [161,162]. Therefore, *S. marcescens* is a potential indicator of environmental pollution, including metal pollution, organic pollution, and fecal contamination [163,164,165]. Many researchers are now focusing on the bioremediation potential of *S. marcescens*, exploring its applications in mitigating metal and organic pollution [162,166]. The abnormal abundance of *S. marcescens* in this study may reflect significant pollution stress in the water body. Among the top 30 most abundant bacterial species, *E. coli*, a well-known indicator of fecal pollution [167], and *Aeromonas caviae*, associated with both fecal and organic pollution [168], were also observed.

In this study, Actinobacteria, with a relative abundance of 2.9–15.8%, is known to correlate with nutrient levels in water bodies [156,169,170]. It is also the dominant microbial phylum in oligotrophic to mesotrophic lakes, with its abundance decreasing as nutrient levels increase [156,169,170]. Bacteroidota, with relative abundances of 4.7–8.1%, like Proteobacteria, are key indicator bacteria for heavy metal and fecal pollution, with their abundance increasing as pollution levels rise [171,172].

In summary, it can be concluded that eutrophication, fecal contamination, and metal pollution may exist in this area, potentially caused by domestic, agricultural, or industrial discharges. However, since bacterial taxa vary between rivers, it is not accurate to measure their abundance in a standardized way. The degree and specific sources of contamination can be further determined by monitoring water quality.

### 4.4. Eukaryotic Community Patterns Indicate Pollution

In the eukaryotic community structure analysis based on 18S rRNA, Ciliophora, Arthropoda, and Chlorophyta were the dominant taxa across all samples, though their relative abundances varied significantly among them. These findings are similar to those of a previous study on eukaryotic community structure in the Xiaoqing River, China [173].

Ciliophora is widespread and abundant in the environment, likely due to its greater tolerance of extreme conditions [174,175]. Numerous studies have also shown that Ciliophora is sensitive to changes in nutrients like nitrogen and phosphorus, making it an effective bioindicator of freshwater quality [176,177,178]. For example, in the Krka River in Croatia, Stentor and Loxophyllum were found in abundance, indicating a higher accumulation of organic matter in the region [179]. Similarly, studies on Brazilian rivers found *Caenomorpha* sp. and *Spirostomum teres* to be sensitive to organic pollution, predominantly present in heavily polluted areas, making them useful as indicator species [180]. In this study, *Strombidium* showed the highest relative abundance (11.8%) at the genus level, demonstrating absolute dominance. It is commonly found in coastal areas [181,182,183] but has a global distribution [184], with particularly high abundance in polluted, eutrophic waters rich in nutrients [178,184]. *Strombidium* exhibits mixotrophic metabolism, allowing it to feed on both algae and bacteria, while obtaining organic matter through predation and retaining prey chloroplasts for photosynthesis [185,186]. A study found that *Strombidium* is highly sensitive to nutrient levels, with its abundance significantly increasing in environments enriched with total nitrogen (TN), ammonium nitrogen (NH_4_-N), and other nutrients and organic matter [187]. Similarly, studies by Basuri and Liu et al. have demonstrated a positive correlation between the abundance of *Strombidium* and environmental factors such as salinity, chlorophyll-a, and nutrient concentrations, suggesting its potential as an ecological indicator for water quality monitoring [188,189]. The dominance of *Strombidium* in the environment may indicate issues related to waterbody eutrophication. In this study, the sampling site was located at a brackish water junction, where elevated salinity levels also contributed to *Strombidium* becoming the dominant genus. However, *Strombidium* was not among the top 30 species by relative abundance, suggesting greater species diversity within this genus. Only three species, led by *Tintinnidium* sp. (relative abundance around 2%), appeared in the top 30 for the entire Ciliophora group.

Arthropoda was significantly dominant at sampling sites P1 and P10, and its relative abundance in P6 and P9 was comparable to that of Ciliophora. *Bestiolina similis* within this clade had a relative abundance of nearly 8%, which is about twice that of the second most abundant species. This species is widely distributed in tropical and subtropical waters and is particularly abundant in areas with transitional salinities [190], such as the Amazon estuary [191]. The sampling area in this study is located at the lower end of the Perak River, near its confluence with the Strait of Malacca, which explains the higher abundance observed. However, the relative abundance reached 39.4% at site P1 and 18.9% at site P10. In a study of species diversity in tropical estuaries, the Perequê-Açu estuary showed a higher abundance of *Bestiolina similis* (19.52%) due to elevated pollution levels [190]. Therefore, based on this indicator, it can be inferred that pollution may be higher at these two sampling sites.

### 4.5. eDNA-NGS Significantly Improves Pathogen Surveillance Capabilities

In this study, potential pathogens identified from 16S rRNA and 18S rRNA sequencing results were classified into three categories: bacteria, fungi, and parasites. Most of the pathogens detected in the Perak River were opportunistic, such as *Serratia marcescens* [80], *Acinetobacter baumannii* [47], and *Klebsiella pneumoniae* [64], etc. Most of these pathogens do not cause disease in immunocompetent individuals. However, in hospital settings or among immunocompromised populations (e.g., the elderly, children, and immunosuppressed patients), they can lead to severe opportunistic infections [192]. These pathogens can enter drinking water systems through rivers and pose a direct risk to humans. Although water sources are treated by purification plants, contamination of drinking water remains a persistent challenge. For example, studies have shown that parasitic pathogens such as *Giardia* and *Acanthamoeba* can still be detected in treated water from certain drinking water treatment plants in Malaysia [193]. In rural Malaysia, drinking water contamination is a major cause of *Blastocystis* infection in children [194]. *Blastocystis* was also detected in the Perak River in this study. Although its relative abundance is low, it can serve as an early warning sign of potential risk.

eDNA sequencing technology can quickly and efficiently identify potential pathogens in the environment, providing essential information for the preliminary identification of pathogens. However, eDNA-NGS method cannot monitor pathogen concentrations, making it difficult to assess whether they pose a public health risk. The combination of qPCR and eDNA-NGS could be a future application to accurately quantify pathogen abundance, allowing for public health risk assessments using infection thresholds such as ID50. In more contaminated rural areas, testing water sources like the Perak River can offer data to help public health departments take early action to prevent pathogen levels from reaching health-threatening thresholds.

In addition, environmental factors significantly impact the spread and transmission of pathogens. Improper discharge of domestic sewage and industrial wastewater can lead to organic and inorganic contamination of water bodies, introducing pathogens and altering the microbial community structure of rivers, which may result in the enrichment of pathogenic microorganisms [195]. During the rainy season, increased rainfall and more frequent storms can raise pathogen concentrations by introducing more pollutants and pathogens into water bodies through surface runoff [196,197]. Higher water temperatures also affect the survival and reproduction of pathogens, with bacteria like *Vibrio* more likely to proliferate in warmer waters [198,199]. Therefore, regular pathogen monitoring in water bodies can help predict the effects of climate change on pathogen abundance and enable the development of preventive measures in advance.

### 4.6. Strengthening Environmental Policies and Public Health with eDNA Insights

We further emphasize the critical role of eDNA-based monitoring programs in water system management. In this study, the high abundance of Gammaproteobacteria and *Serratia marcescens*, as well as the positive correlation between *Strombidium* abundance and organic matter concentration, suggest the potential presence of eutrophication and other pollution issues in the sampling area. Regular biodiversity surveys allow for real-time tracking of microbial diversity changes and rapid identification of potential pollution sources, without the need for comprehensive monitoring of all contaminants [200]. Conducting inspections of agricultural lands and industrial discharge areas near the river can effectively identify pollution sources, providing a scientific basis for resource allocation and remediation measures. Additionally, improving wastewater management, such as upgrading treatment facilities or establishing vegetative buffer zones, can reduce nutrient and pollutant inputs, thereby mitigating the impacts of eutrophication on aquatic ecosystems [201,202]. These measures help protect the health of river ecosystems and promote sustainable environmental management.

Meanwhile, the findings of this study also provide significant applications in the field of public health. The pathogens detected in this study (as shown in Table 2, Table 3 and Table 4), including *Serratia marcescens* and other bacteria, fungi, and parasites, are largely associated with human diseases such as septicemia and gastrointestinal infections. Moreover, outbreaks of certain pathogens may cause mass mortality in wildlife populations, thereby threatening the stability of the ecosystem. The eDNA-based screening technology provides comprehensive information on potential pathogens in the environment, which is of significant importance for early disease warning and risk management [203,204]. This study identified *Serratia marcescens* as the most abundant pathogen, suggesting a potentially high public health risk in the sampling area. To address this issue, it is recommended that nearby water treatment plants enhance filtration and disinfection processes, with particular focus on pathogens of high abundance. Additionally, residents should be advised to use boiled water to reduce infection risks. These targeted measures not only mitigate the threat of pathogens to human health but also contribute to improving the health and stability of aquatic ecosystems.

However, there remains room for further improvement in future research. Firstly, although this study provides valuable ecological data, its temporal dimension is relatively limited. Conducting biodiversity monitoring on a regular basis, such as quarterly, could more comprehensively capture the ecological dynamics within the river system that arise from seasonal variations. This would enable a deeper understanding of how factors such as temperature, water levels, and other environmental conditions influence the structure of microbial communities [205,206].

Secondly, the relationship between biodiversity and environmental pollution may vary significantly across different river systems, necessitating further exploration of the validity and applicability of regional ecological indicators. Future research could focus on developing a set of standardized biological indicators linked to specific pollution levels for the Perak River system. For instance, correlating the abundance of certain dominant genera with specific pollutants, such as organic matter, heavy metals, or nitrogen and phosphorus compounds, could enable rapid pollution assessment by monitoring biodiversity [207,208,209]. This approach could significantly enhance the efficiency of environmental management while reducing reliance on complex physicochemical analyses, ultimately saving resources and costs.

Moreover, the application of eDNA technology in pathogen screening requires further development. This study has identified several potential pathogens, but integrating eDNA with qPCR for precise quantification of pathogen abundance and comparing it to infection thresholds [210] would enable a more accurate assessment of their potential public health risks. In conclusion, future research should aim to enhance monitoring frequency, establish targeted ecological indicator systems, and refine quantitative methodologies to provide more scientific and efficient solutions for the sustainable management of river systems such as the Perak River and the protection of public health.

## 5. Conclusions

This study used metabarcoding and eDNA-NGS technologies to examine the biodiversity and community composition of eukaryotes and bacteria in the Perak River, Malaysia. By comparing these results with past studies in both natural and polluted environments, we made an initial assessment of the river’s health. Additionally, eDNA-NGS helped detect potential pathogens in the river, which can alert local authorities to possible health risks. This early detection is important for monitoring water quality and preventing disease outbreaks in communities that rely on the river. In the future, combining this method with other tools can improve our ability to measure pathogen levels and better protect public health.

## Figures and Tables

**Table 1:** Sampling Locations and Coordinates in the Perak River Basin.

Location ID	Name of Location	Coordinates	Sample ID
L1	Sungai Dulang Dalam	3°57′29.2″ N 100°52′35.9″ E	P1, P2, P3
L2	Sungai Dulang	3°57′32.8″ N 100°52′03.7″ E	P4, P5, P6
L3	Kota Setia	4°01′25.0″ N 100°52′10.3″ E	P7, P8, P9
L4	Bagan Datuk	3°59′33.7″ N 100°47′08.0″ E	P10, P11, P12
L5	Beting Beras Basah	4°00′15.9″ N 100°43′23.7″ E	P13, P14, P15

## Data Availability

The original contributions presented in this study are included in the article/Supplementary Materials. Further inquiries can be directed to the corresponding authors.

## Supplementary Materials

The following supporting information can be downloaded at: https://www.mdpi.com/article/10.3390/microorganisms13092055/s1, Table S1: Summary of 16S and 18S rRNA Amplicon Sequencing Statistics; Table S2: 16S and 18S rRNA Amplicon Sequence Length Distribution Summary; Table S3: 16S and 18S rRNA OTU Abundance Table Across Samples.
